# Validation of a point-of-care handheld blood total calcium analyzer in postpartum dairy cows

**DOI:** 10.3168/jdsc.2020-0006

**Published:** 2020-12-11

**Authors:** Rita Couto Serrenho, Tony C. Bruinjé, Emma I. Morrison, David L. Renaud, Trevor J. DeVries, Todd F. Duffield, Stephen J. LeBlanc

**Affiliations:** 1 Population Medicine, University of Guelph, Guelph, ON, Canada N1G 2W1; 2 Animal Biosciences, University of Guelph, Guelph, ON, Canada N1G 2W1

## Abstract

Our objective was to validate a point-of-care handheld blood total calcium analyzer (Ca meter, CM; TD-5220 Vet Ca^2+^, TaiDoc, New Taipei, Taiwan) to estimate circulating Ca concentrations in postpartum dairy cows. Whole blood was collected from 251 multiparous cows between 1 and 4 d in milk from 2 commercial dairy herds in Ontario, Canada. Blood total calcium concentration (tCa) was analyzed in whole blood, fresh plasma, and thawed plasma, and compared with tCa results from thawed serum analyzed in a diagnostic laboratory (using a Cobas Calcium Gen 2 kit, Roche Diagnostics, Indianapolis, IN) as the reference test (RT). Lin's concordance correlation coefficient (βrho;) and Bland-Altman (B-A) plots were assessed to evaluate the agreement between the RT and CM results in each type of sample. Receiver operating characteristic curve analyses were used to describe the accuracy of each test against the categorized RT results (at a cut-point of ≤2.14 mmol/L). Samples where the meter gave a nonquantitative result (“high” or “low”; thawed plasma: 3/247; fresh plasma: 6/100; and whole blood: 20/98) were not included in the βrho; and B-A analyses. Lin's correlation coefficients demonstrated poor agreement between tests (thawed plasma: βrho; = 0.16; fresh plasma: βrho; = 0.21; and whole blood: βrho; = 0.23). Fresh plasma (using a cut-point of 2.55 mmol/L as measured on the CM) had the greatest diagnostic sensitivity (72%), specificity (86%), and accuracy (77%) for determining subclinical hypocalcemia, but that would still misclassify 23% of samples. In addition to substantial variability, the B-A plots revealed bias with changing concentrations of calcium. Because of low sensitivity on whole blood (58%) or thawed plasma (56%), measurement with the CM is not recommended on these types of samples. This rapid and low-cost meter was not sufficiently accurate to quantify blood Ca concentration, but when used with fresh plasma it might be useful as a screening tool for subclinical hypocalcemia.

## Highlights

• It is desirable to identify cows for selective administration of Ca supplements
• The objective was to validate a cow-side meter to estimate blood Ca in dairy cows
• The meter was not sufficiently accurate to quantify blood Ca concentration
• The meter might be useful to classify subclinical hypocalcemia in fresh plasma

## Graphical Abstract

**Figure:**
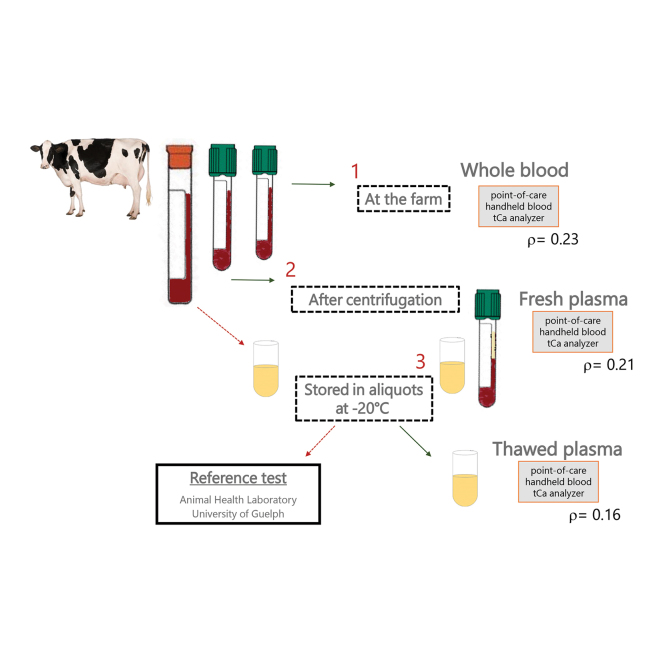
**Summary:** Our objective was to validate a point-of-care handheld blood calcium analyzer to estimate circulating calcium concentrations in postpartum dairy cows. An accurate, rapid, and inexpensive tool to screen for hypocalcemia on-site would allow for implementation of selective treatment protocols but the meter assessed was not sufficiently accurate to quantify blood calcium concentration.

More than 50% of multiparous cows experience subclinical hypocalcemia (**SCH**) within the first 4 d after parturition (Martinez et al., 2012; McArt and Neves, 2020). The cut-point to define SCH varies among studies, depending on the outcome of interest, and when postpartum and how many samples per cow were assessed. Subclinical hypocalcemia is associated with increased risk of metritis and other postpartum diseases (Chapinal et al., 2011; Martinez et al., 2012; Rodríguez et al., 2017). It may also be associated with reduced milk yield in early lactation, depending on when blood Ca is measured and the duration of SCH (Chapinal et al., 2012; Neves et al., 2018a; McArt and Neves, 2020). Cows with blood total Ca (**tCa**) ≤2.14 mmol/L in the first 3 DIM tend to have lower pregnancy rates and to take longer to return to cyclicity (Martinez et al., 2012; Caixeta et al., 2017). Because SCH is common and associated with undesirable outcomes, peripartum Ca supplementation is a common practice in dairy herds (USDA-NAHMS, 2018).

However, the effects of postpartum Ca supplementation appear to depend on parity, milk production potential, or pretreatment tCa. In multiparous cows, postpartum Ca supplementation enhanced reproductive performance, and increased milk yield in cows with milk yield greater than their herd average in the previous lactation, but decreased production in cows with below-average production (Martinez et al., 2016b). Calcium supplementation reduced disease risk in multiparous cows with lower blood Ca concentration (Leno et al., 2018) or those with excessive BCS or pre-existing lameness (Oetzel and Miller, 2012; Leno et al., 2018). In primiparous cows, Ca supplementation reduced pregnancy at first insemination and increased the risk of postpartum disease when tCa at parturition was >2.15 mmol/L (Martinez et al., 2016a,b; Leno et al., 2018). Therefore, it would be desirable to selectively treat multiparous cows likely to benefit from Ca supplements. An accurate, rapid, and inexpensive tool to screen for SCH on-site would allow for implementation of treatment protocols based on circulating blood Ca concentration at specific days in milk and to avoid blanket treatments that could incur costs without benefits.

To date, only one portable clinical analyzer has been validated for hypocalcemia testing in cattle (Peiró et al., 2010). However, its purchase cost and the cost of individual cartridges represent a barrier to its routine use on farm. Therefore, the objective of this cross-sectional diagnostic accuracy study was to validate a point-of-care handheld blood total Ca analyzer (TD-5220 Vet Ca^2+^, TaiDoc, New Taipei, Taiwan; calcium meter, **CM**) to estimate circulating Ca concentration in postpartum, multiparous dairy cows.

This work is reported using the Standards for Reporting of Diagnostic Accuracy Studies (STARD) Guideline (Cohen et al., 2016). Sample collection was approved by the University of Guelph Animal Care Committee (Guelph, ON, Canada; AUP# 3951). Whole blood was collected from the coccygeal vessels from 251 multiparous cows at 1, 2, 3, or 4 DIM (1 sample per cow) between September 2019 and February 2020 from 2 commercial dairy herds in Ontario, Canada, milking approximately 450 (farm A) or 400 (farm B) Holstein cows.

Blood was collected with a 22-gauge, 1.5-inch needle into a 10-mL tube without anticoagulant (BD Vacutainer Precision Glide; Becton Dickinson, Franklin Lakes, NJ) and into 4-mL and 6-mL sodium heparinized tubes (Vacutainer, Becton Dickinson). All testing with the CM was performed following the manufacturer's guidelines. In short, 0.5 mL of air followed by 0.5 mL of the sample were drawn into a 1-mL syringe; a small plastic filter (provided by the manufacturer) was attached to a syringe; a test strip was introduced into the meter; and a drop of filtered blood or fresh or thawed plasma was placed in the strip well. After 180 s, a result was displayed on the screen.

Within 60 min of sampling, a sample of heparinized whole blood (4-mL tube) was tested at the farm with the CM, indoors at approximately 22°C. The 6-mL heparinized sample tube was placed on ice and the 10-mL whole blood sample was maintained at room temperature until processing in the laboratory. Within 4 h of sampling, the 6-mL and 10-mL tubes were centrifuged at 1,500 × *g* at room temperature for 15 min. A sample of the fresh plasma was used to measure tCa with the CM as previously described, while the serum and remaining plasma were stored in aliquots at −20°C for further analysis. Frozen plasma was thawed at room temperature and tested with the CM, whereas frozen serum was sent to the Animal Health Laboratory (University of Guelph, ON, Canada) for measurement of tCa using a Cobas Calcium Gen 2 kit (Roche Diagnostics, Indianapolis, IN), which was considered the reference test (**RT**). Although atomic absorption spectrophotometry is considered the gold standard to measure calcium concentration, photometric methods are commonly used in clinical practice (Kimura et al., 1996; Bazydlo et al., 2014). These validated methods are available at a lower cost (Kimura et al., 1996; Bourguignon et al., 2014).

The CM consisted of a colorimetric method to estimate blood tCa concentration between 0.5 and 3.0 mmol/L in increments of 0.1 mmol/L, with outputs of “low” or “high” if values were out of the lower or upper limits of quantification. The manufacturer provided instructions to test tCa in heparinized whole blood and fresh plasma (sample size of 45 µL). Because serum samples were frozen and thawed before being tested at the Animal Health Laboratory, we also tested thawed plasma. The RT is a photometric method with a range of quantification of 0.20 to 5.0 mmol/L, and intra- and interassay coefficients of variation of 1.3 and 2.3%, respectively. Independent of the samples used to assess the CM, we compared tCa measured by the RT method in 20 paired plasma and serum samples from the same cows at the same time (blood collected into tubes with lithium heparin or no anticoagulant, respectively). Regression of these data gave the relationship serum Ca = 0.373 × 0.812(plasma Ca); R^2^ = 0.94, βrho; = 0.92, with a mean difference of 0.05 ± 0.06 mmol/L, where βrho; is Lin's concordance correlation coefficient.

Sample size was calculated using the method described by Buderer (1996). A prevalence of SCH (defined as serum concentration ≤2.14 mmol/L) of at least 50% was used based on previous studies in multiparous cows (Martinez et al., 2012; Caixeta et al., 2017). To provide a conservative estimate, we calculated the sample size using expected sensitivity (**Sn**) and specificity (**Sp**) of 50%, and a clinically acceptable width of the 95% CI for Sn and Sp of 10%, requiring a minimum of 193 samples.

Assumptions of normality were assessed with the Shapiro-Wilk test and histograms for all continuous variables. Data from the CM results were censored at <0.5 and >3.0 mmol/L, which compromised its normal distribution. Transformations of the data did not improve the distribution (negatively skewed) so the data were not transformed. Lin's concordance correlation coefficient (**βrho;**; Lin, 1989) was calculated using the macro “CCC V9” (Crawford et al., 2007) in SAS version 9.4 (SAS Institute Inc., Cary, NC) to measure the agreement between the RT (thawed serum) and each of the CM results (whole blood, fresh plasma, or thawed plasma). To evaluate bias between each CM test and the RT, Bland-Altman (**B-A**) plots (Bland and Altman, 1986) were generated in SAS. For calculation of βrho; and the B-A plots, values reported as “high” or “low” by the CM were excluded. Graphical representation of Lin's regressions and the B-A plots were created using Excel (Windows 10, version 1903; Microsoft Corp., Redmond, WA). Total calcium values from RT were categorized as SCH (≤2.14 mmol/L; Martinez et al., 2012) or normocalcemia (**NC**; >2.14 mmol/L). Contingency tables were created to calculate Sn, Sp, positive and negative predictive values, and accuracy (percentage of samples correctly classified). Receiver operating characteristic (**ROC**) curve analysis was performed using PROC LOGISTIC in SAS to calculate the area under the curve and identify the threshold with highest combined Sn and Sp (the point on ROC curve closest to x = 0 and y = 1); false negatives and false positives were weighted equally. For all categorical analyses, “high” and “low” readings from the CM were replaced by 3 or 0.5 mmol/L, respectively, and included as classifications above or below the cut-point of interest. Because of damage to the CM at the beginning of the study, fewer samples were analyzed as whole blood or fresh plasma than as thawed plasma (Table 1). While we waited for a new meter, blood sample collection continued, providing frozen plasma but no fresh samples. We repeated all the statistical analyses above after applying a correction to the serum data based on the regression of serum versus plasma tCa measured with the RT.

**Table 1:** Descriptive statistics of blood total calcium concentrations (tCa) on d 1, 2, 3, or 4 after calving from 251 multiparous cows, measured with a point-of-care meter or in a diagnostic laboratory (reference test)

Variable	Point-of-care calcium analyzer^1^, ^2^			Reference test^3^
	Whole blood	Fresh plasma	Thawed plasma	Thawed serum
Samples (no.)	78	94	247	251
“High”	9	5	3	—
“Low”	11	1	0	—
Blood tCa (mmol/L)^4^				
Mean (SD)	2.3 (0.6)	2.5 (0.2)	2.5 (0.3)	2.03 (0.29)
Median (IQR)^5^	2.5 (0.8)	2.5 (0.3)	2.5 (0.3)	2.05 (0.38)
Maximum	3.0	3.0	3.0	2.63
Minimum	0.5	1.9	1.6	1.05

1 TD-5220 Vet Ca^2+^ (TaiDoc, New Taipei, Taiwan).

2 All samples were filtered with a device provided by the manufacturer.

3 Cobas Calcium Gen 2 kit (Roche Diagnostics, Indianapolis, IN).

4 Samples with “high” (>3.0 mmol/L) or “low” (<0.5 mmol/L) readings from the calcium analyzer were not included.

5 Interquartile range.

A total of 251 samples from multiparous cows within the first 4 DIM were included in this study. During the time that the CM was not functional, 153 whole blood and 151 fresh plasma samples were not tested. The calculation of βrho; and analysis of B-A plots did not include 3, 6, and 20 thawed plasma, fresh plasma, and whole blood samples, respectively, due to the lack of a numeric result from the CM. The prevalence of SCH (≤2.14 mmol/L) using the RT was 63.7% [farm A: 73.8% (96/130); farm B: 52.9% (64/121)]. Descriptive statistics are presented in Table 1. Lin's correlation coefficient demonstrated poor agreement between the CM and RT measurement for blood tCa concentration (thawed plasma: βrho; = 0.16; fresh plasma: βrho; = 0.21; and whole blood: βrho; = 0.23; with the plasma vs. serum regression applied to the data, βrho; = 0.18, 0.24, and 0.26, respectively). The inferences were the same for all the following analyses whether or not we adjusted the serum values, so unadjusted data are presented here. Most of the data points are located above the line of perfect agreement, indicating overestimation by the CM (Figure 1a). The same is indicated by the B-A plots, where the negative bias present in all 3 plots (Figure 1b) indicated that the CM values were greater than the RT for the same sample. For thawed plasma, fresh plasma, and whole blood, these plots showed a mean difference (bias) of −0.44, −0.53, and −0.26 mmol/L, respectively. This means that, on average, the CM gave a result on thawed plasma that was 0.44 mmol/L ± 0.31 greater than the RT value. The B-A plots also illustrated random variability between the results from the CM and RT. Although most of the data fall within the confidence limits on the B-A plots, that does not indicate that differences within these bounds are biologically or practically acceptable. Moreover, mean errors of 0.26 to 0.53 mmol/L are meaningful differences when measuring tCa postpartum. For context, the allowable error for measurement of tCa in human samples is ±0.25 mmol/L (Verma et al., 2019). The variability between CM and RT appeared to be randomly distributed for both fresh and thawed plasma but not for whole blood. The B-A plots demonstrated a positive bias with low mean tCa concentrations (i.e., the RT result was higher) and a negative bias with a greater mean tCa concentration. It is possible that the fewer samples in the low tCa range contributed to this difference. However, this should be taken into consideration because this meter is recommended for use with whole blood. The large mean bias with substantial variability and low concordance between tests led us to conclude that the CM results, on a continuous scale, were not good estimates of tCa.

**Figure 1:**
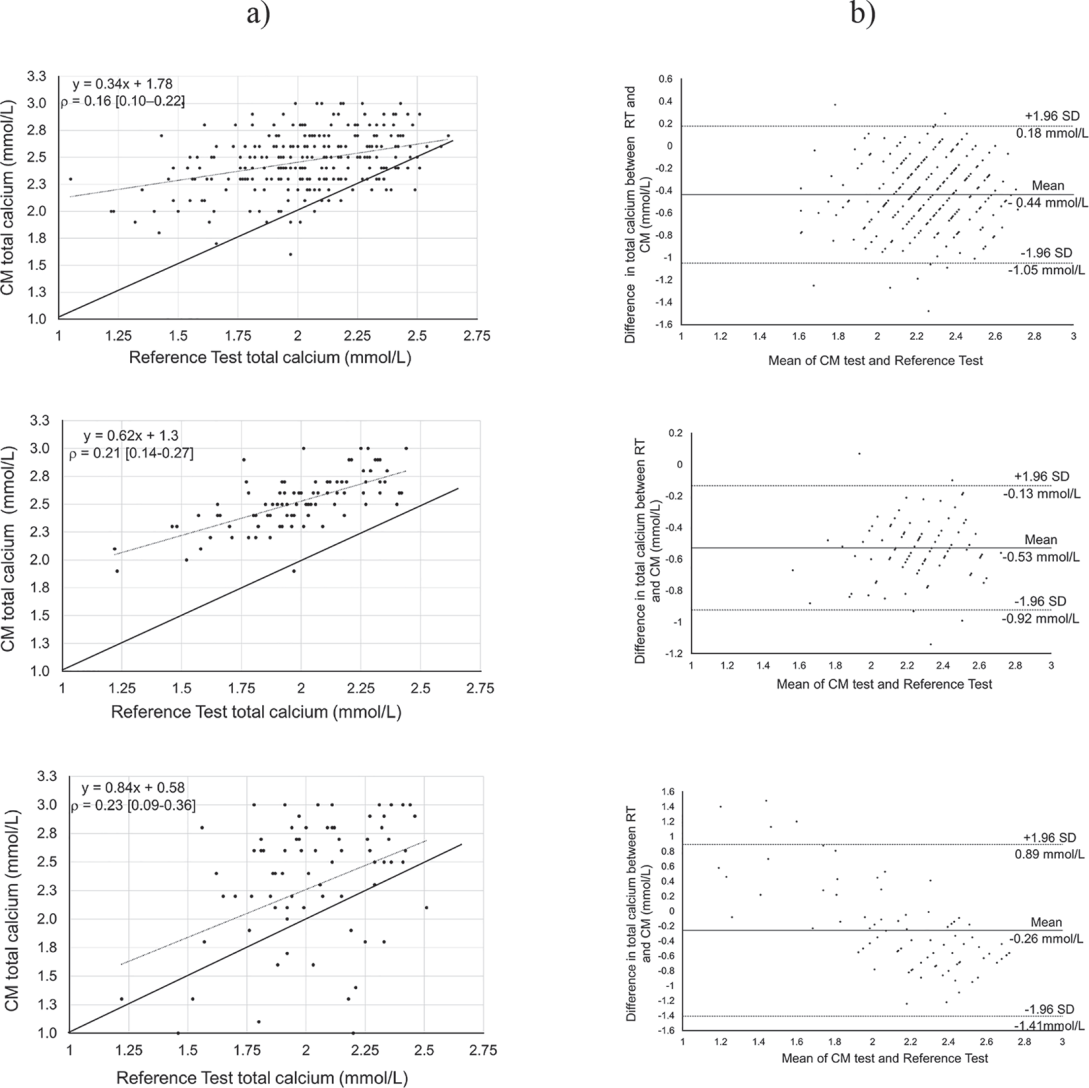
Agreement between a point-of-care calcium analyzer (CM; TD-5220 Vet Ca^2+^, TaiDoc, New Taipei, Taiwan) used with thawed plasma (top row), fresh plasma (middle row), or whole blood (bottom row) and serum total Ca concentration measured in a diagnostic laboratory (reference test, RT; Cobas Calcium Gen 2 kit, Roche Diagnostics, Indianapolis, IN). (a) Regression showing Lin's concordance correlation coefficient (βrho;) and its 95% CI; the solid line represents perfect concordance, and the dashed line represents the observed concordance regression. (b) Bland-Altman plot of differences between the CM and the mean of the results from the CM and the reference test. For thawed plasma (top row), there were 247 samples (1 sample per cow between 1 and 4 DIM), excluding 3 for which no numeric result was provided by the CM. For fresh plasma (middle row), there were 94 samples (1 sample per cow between 1 and 4 DIM), excluding 6 for which no numeric result was provided by the CM. For whole blood (bottom row), there were 78 samples (1 sample per cow between 1 and 4 DIM), excluding 20 for which no numeric result was provided by the CM.

The main goal of a point-of-care, cow-side Ca meter is to discriminate usefully between cows with and without SCH. This would prevent unnecessary administration of Ca supplementation to cows with NC. Categorization of RT tCa results into SCH or NC allowed us to assess the test characteristics of the CM (Table 2). Our results demonstrated that with accuracy of 60% for thawed plasma or whole blood, the CM did not perform well enough to classify individual cows by calcemia status. Although the CM was designed to be used directly on whole blood with a filtration device provided with the meter, our results do not support the use of the CM with whole blood or thawed plasma. Moreover, approximately 25% of the whole blood samples tested did not provide a numeric result. This would meaningfully reduce the utility of the meter.

**Table 2:** Test characteristics of a point-of-care calcium analyzer (CM; TD-5220 Vet Ca^2+^, TaiDoc, New Taipei, Taiwan) relative to serum total calcium measured in a diagnostic laboratory (reference test; Cobas Calcium Gen 2 kit, Roche Diagnostics, Indianapolis, IN)

Sample measured by CM^1^	Cut-point for CM^2^ (mmol/L)	Sensitivity^3^ (%)	Specificity^4^ (%)	PPV^5^(%)	NPV^6^ (%)	Accuracy^7^ (%)
Thawed plasma (n = 250)	2.45	56	66	74	46	60
Fresh plasma (n = 100)	2.55	72	86	90	63	77
Whole blood (n = 98)	2.40	58	64	73	45	60

1 Whole blood, and fresh and thawed plasma samples were collected from multiparous cows at d 1, 2, 3, or 4 postpartum.

2 Based on receiver operator characteristics curve analysis.

3 The probability of being classified as hypocalcemic using the CM given that the cow had a blood total Ca concentration (tCa) ≤2.14 mmol/L as measured by the reference test.

4 The probability of being classified as not hypocalcemic using the CM given that the cow had a blood tCa >2.14 mmol/L as measured by the reference test.

5 Positive predictive value = the probability that a cow had tCa ≤2.14 mmol/L given that the cow was classified as hypocalcemic by the CM. The prevalence of subclinical hypocalcemia (SCH; ≤2.14 mmol/L) using the reference test was 63.7%.

6 Negative predictive value = the probability that a cow had tCa >2.14 mmol/L given that the cow was classified as not hypocalcemic by the CM. The prevalence of SCH (≤2.14 mmol/L) using the reference test was 63.7%.

7 Proportion of correctly classified blood samples (true positives + true negatives) among all tested blood samples.

The CM performed better with fresh plasma (using a cut-point of 2.55 mmol/L), with 77% accuracy. A positive predictive value of 90% means there would be relatively few errors of falsely classifying cows as hypocalcemic. Conversely, the moderate sensitivity indicates a 28% chance that a SCH case would be missed. Due to the relatively high specificity, the CM might be useful if it were important to avoid the cost of treating NC cows with a calcium supplement. However, as used here, the fresh plasma sample required time and equipment to separate plasma in a blood collection tube in a centrifuge. Nevertheless, the turnaround time and the cost per sample would be less than sending a blood sample to a diagnostic laboratory.

Because the first CM was damaged, a considerable number of whole blood and fresh plasma samples were not tested, causing possible information bias. However, given the poor performance demonstrated in the acquired samples, it is unlikely that more samples would change the conclusion. Thawed plasma samples were only assessed with the second meter, whereas whole blood and fresh plasma were assessed with both. The differences in the mean values could be confounded by differences between the instruments. If such systematic error exists between meters, that would, in any case, limit the utility of the tool.

Fresh and thawed plasma in the CM were compared with serum in the RT. The laboratory running the RT specifies use of serum because their reference intervals are based on serum samples. However, for the CM, the manufacturer recommends the use of plasma or whole blood samples. Our data comparing paired plasma and serum samples using the RT showed a mean difference of 0.05 mmol/L. Bach et al. (2020) assessed blood tCa concentrations in serum and plasma over time, showing an average difference of 0.03 mmol/L Ca between serum and plasma (serum: 2.25 mmol/L; plasma: 2.28 mmol/L). To account for the difference between serum and plasma tCa, a correction factor could be applied. However, our inferences would remain the same because the mean biases observed in the B-A plots were 5- to 10-fold greater than the mean difference between serum and plasma.

Based on low concordance with the RT, we conclude that the CM was not sufficiently accurate to quantify tCa concentration. However, with an adjusted cut-point, when used with fresh plasma, with sensitivity of 72% and specificity of 86%, it might be useful as a screening tool for SCH. The performance of the CM for on-farm assessment of tCa with whole blood was not adequate to select cows to receive a Ca supplement after calving.

## References

[bib1] Bach K.D., Neves R.C., Stokol T., McArt J.A.A. (2020). Technical note: Effect of storage time and temperature on total calcium concentrations in bovine blood. J. Dairy Sci..

[bib2] Bazydlo L.A.L., Needham M., Harris N.S. (2014). Calcium, magnesium, and phosphate. Lab. Med..

[bib3] Bland J.M., Altman D.G. (1986). Statistical methods for assessing agreement between two methods of clinical measurement. Lancet.

[bib4] Bourguignon C., Dupuy A.M., Coste T., Michel F., Cristol J.P. (2014). Evaluation of NM-BAPTA method for plasma total calcium measurement on Cobas 8000®. Clin. Biochem..

[bib5] Buderer N.M.F. (1996). Statistical methodology: I. Incorporating the prevalence of disease into the sample size calculation for sensitivity and specificity. Acad. Emerg. Med..

[bib6] Caixeta L.S., Ospina P.A., Capel M.B., Nydam D.V. (2017). Association between subclinical hypocalcemia in the first 3 days of lactation and reproductive performance of dairy cows. Theriogenology.

[bib7] Chapinal N., Carson M., Duffield T.F., Capel M., Godden S., Overton M., Santos J.E.P., LeBlanc S.J. (2011). The association of serum metabolites with clinical disease during the transition period. J. Dairy Sci..

[bib8] Chapinal N., Carson M.E., LeBlanc S.J., Leslie K.E., Godden S., Capel M., Santos J.E.P., Overton M.W., Duffield T.F. (2012). The association of serum metabolites in the transition period with milk production and early-lactation reproductive performance. J. Dairy Sci..

[bib9] Cohen J.F., Korevaar D.A., Altman D.G., Bruns D.E., Gatsonis C.A., Hooft L., Irwig L., Levine D., Reitsma J.B., De Vet H.C.W., Bossuyt P.M.M. (2016). STARD 2015 guidelines for reporting diagnostic accuracy studies: Explanation and elaboration. BMJ Open.

[bib10] Crawford S.B., Kosinski A.S., Lin H.M., Williamson J.M., Barnhart H.X. (2007). Computer programs for the concordance correlation coefficient. Comput. Methods Programs Biomed..

[bib11] Kimura S., Iyama S., Yamaguchi Y., Hayashi S., Fushimi R., Amino N. (1996). New enzymatic assay for calcium in serum. Clin. Chem..

[bib12] Leno B.M., Neves R.C., Louge I.M., Curler M.D., Thomas M.J., Overton T.R., McArt J.A.A. (2018). Differential effects of a single dose of oral calcium based on postpartum plasma calcium concentration in Holstein cows. J. Dairy Sci..

[bib13] Lin L.I.-K. (1989). A concordance correlation coefficient to evaluate reproducibility. Biometrics.

[bib14] Martinez N., Risco C.A., Lima F.S., Bisinotto R.S., Greco L.F., Ribeiro E.S., Maunsell F., Galvão K., Santos J.E.P. (2012). Evaluation of peripartal calcium status, energetic profile, and neutrophil function in dairy cows at low or high risk of developing uterine disease. J. Dairy Sci..

[bib15] Martinez N., Sinedino L.D.P., Bisinotto R.S., Daetz R., Lopera C., Risco C.A., Galvão K.N., Thatcher W.W., Santos J.E.P. (2016). Effects of oral calcium supplementation on mineral and acid-base status, energy metabolites, and health of postpartum dairy cows. J. Dairy Sci..

[bib16] Martinez N., Sinedino L.D.P., Bisinotto R.S., Daetz R., Risco C.A., Galvão K.N., Thatcher W.W., Santos J.E.P. (2016). Effects of oral calcium supplementation on productive and reproductive performance in Holstein cows. J. Dairy Sci..

[bib17] McArt J.A., Neves R. (2020). Association of transient, persistent, or delayed subclinical hypocalcemia with early lactation disease, removal, and milk yield in Holstein cows. J. Dairy Sci..

[bib18] Neves R.C., Leno B.M., Bach K.D., McArt J.A.A. (2018). Epidemiology of subclinical hypocalcemia in early-lactation Holstein dairy cows: The temporal associations of plasma calcium concentration in the first 4 days in milk with disease and milk production. J. Dairy Sci..

[bib19] Oetzel G.R., Miller B.E. (2012). Effect of oral calcium bolus supplementation on early-lactation health and milk yield in commercial dairy herds. J. Dairy Sci..

[bib20] Peiró J.R., Borges A.S., Gonçalves R.C., Mendes L.C.N. (2010). Evaluation of a portable clinical analyzer for the determination of blood gas partial pressures, electrolyte concentrations, and hematocrit in venous blood samples collected from cattle, horses, and sheep. Am. J. Vet. Res..

[bib21] Rodríguez E.M., Arís A., Bach A. (2017). Associations between subclinical hypocalcemia and postparturient diseases in dairy cows. J. Dairy Sci..

[bib22] USDA-NAHMS (2018).

[bib23] Verma S., Redfield R., Azar A.M. (2019). Clinical Laboratory Improvement Amendments of 1988 (CLIA) Proficiency Testing Regulations Related to Analytes and Acceptable Performance - Proposed Changes. Fed. Regist..

